# Follistatin-Like Proteins: Structure, Functions and Biomedical Importance

**DOI:** 10.3390/biomedicines9080999

**Published:** 2021-08-12

**Authors:** Olga K. Parfenova, Vladimir G. Kukes, Dmitry V. Grishin

**Affiliations:** 1 Institute of Biomedical Chemistry (IBMC), 10 Pogodinskaya St., 119121 Moscow, Russia; oparfenova22@gmail.com; 2 Scientific Centre for Expert Evaluation of Medicinal Products, 8/2 Petrovsky Blvd, 127051 Moscow, Russia; gengdv@inbox.ru

**Keywords:** follistatin-like proteins, cellular signaling, biomarkers, cardiovascular disease, cancer, inflammation, respiratory disease, metabolism

## Abstract

Main forms of cellular signal transmission are known to be autocrine and paracrine signaling. Several cells secrete messengers called autocrine or paracrine agents that can bind the corresponding receptors on the surface of the cells themselves or their microenvironment. Follistatin and follistatin-like proteins can be called one of the most important bifunctional messengers capable of displaying both autocrine and paracrine activity. Whilst they are not as diverse as protein hormones or protein kinases, there are only five types of proteins. However, unlike protein kinases, there are no minor proteins among them; each follistatin-like protein performs an important physiological function. These proteins are involved in a variety of signaling pathways and biological processes, having the ability to bind to receptors such as DIP2A, TLR4, BMP and some others. The activation or experimentally induced knockout of the protein-coding genes often leads to fatal consequences for individual cells and the whole body as follistatin-like proteins indirectly regulate the cell cycle, tissue differentiation, metabolic pathways, and participate in the transmission chains of the pro-inflammatory intracellular signal. Abnormal course of these processes can cause the development of oncology or apoptosis, programmed cell death. There is still no comprehensive understanding of the spectrum of mechanisms of action of follistatin-like proteins, so the systematization and study of their cellular functions and regulation is an important direction of modern molecular and cell biology. Therefore, this review focuses on follistatin-related proteins that affect multiple targets and have direct or indirect effects on cellular signaling pathways, as well as to characterize the directions of their practical application in the field of biomedicine.

## 1. Introduction

Despite the rapid development of biomedical technologies, the search for a new candidate drugs and the selection of informative biomarkers involved in significant biological pathways and interactions underlying some pathological processes remains one of the most compelling problems of modern science, as they are able to affect the correct diagnosis and the subsequent choice of a treatment strategy. Within the framework of the above, follistatin-like proteins seem to be interesting objects, since they belong to the major family of acidic cysteine-rich secreted glycoproteins (SPARC) that are highly homologous in both primary sequence and domain structure to the activin-binding protein, follistatin (FST) [1]. These homologous proteins are involved in the modulation of cell interactions with the extracellular milieu.

**Figure 1:**
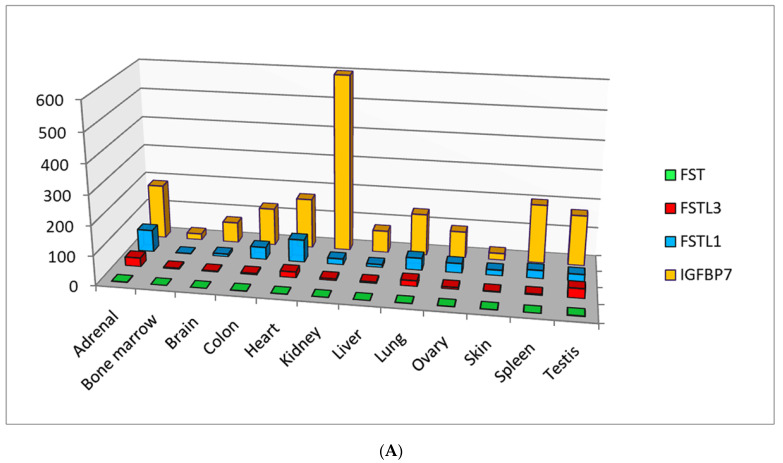

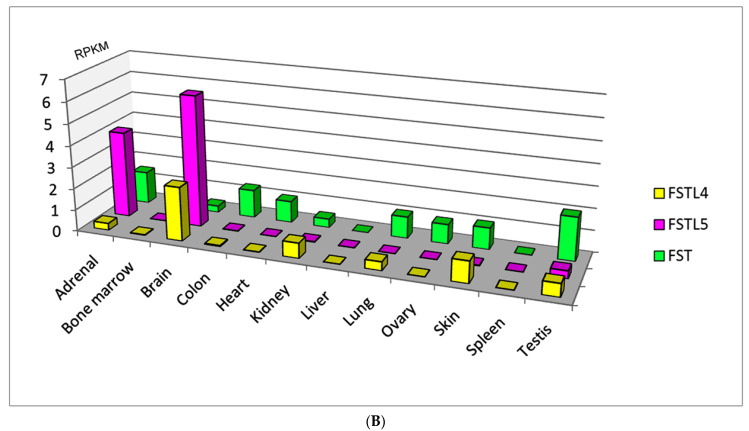
(**A**) Follistatin-like proteins with high tissue-specific expression compared to follistatin (according to the HPA RNA-seq normal tissues project data annotated in the National Center for Biotechnology Information (NCBI) (https://www.ncbi.nlm.nih.gov/, accessed on 1 August 2021)) (RPKM—a normalized unit of transcript expression in reads per kilobase per million of mapped reads). (**B**) Follistatin-like proteins with medium or low tissue-specific expression compared to follistatin (according to the HPA RNA-seq normal tissues project data annotated in the National Center for Biotechnology Information (NCBI) (https://www.ncbi.nlm.nih.gov/, accessed on 1 August 2021)) (RPKM—a normalized unit of transcript expression in reads per kilobase per million of mapped reads).

Nowadays there are five types of follistatin-like proteins: FSTL1, IGFBP7(FSTL2), FSTL3, FSTL4 and FSTL5, having defined the similarities and differences in the domain of classification that represent their specificity [1,2,3]. Homologues of follistatin are expressed in almost all organ systems and tissues (Figure 1), possess the paracrine and autocrine activity, and the nature of their expression changes depending on the severity of pathological processes, including cardiovascular diseases (CVD) [4,5,6,7,8,9], diseases of the respiratory system [10,11,12,13,14,15,16], cancer progression [17,18,19,20,21,22,23,24] and inflammatory diseases [23,25,26,27,28,29]. According to the level of expression in various tissues, follistatin-like proteins can be conditionally divided into proteins with low, medium and high expression compared to that of follistatin (Figure 1A,B). Recent studies have led to the understanding that these proteins are involved in many intracellular signaling pathways [7,24,30,31,32,33,34]. For this reason, follistatin-like proteins and their encoding genes are promising biomarkers from prognostic and diagnostic points of view. Nevertheless, their physiological role and role in the progression of most pathological processes remains unclear and requires deeper understanding and systematization.

This review summarizes the potential role of follistatin-like proteins in the pathogenesis of CVD, inflammatory reactions, respiratory diseases, cancer and metastasis, and disorders of lipid metabolism and the central nervous system (CNS). These systematizations can give a new impetus for future research, prevention and treatment of a variety of diseases.

## 2. Molecular Characteristics of Organization and Transcription of Follistatin-Like Protein Genes

**Figure 2:**
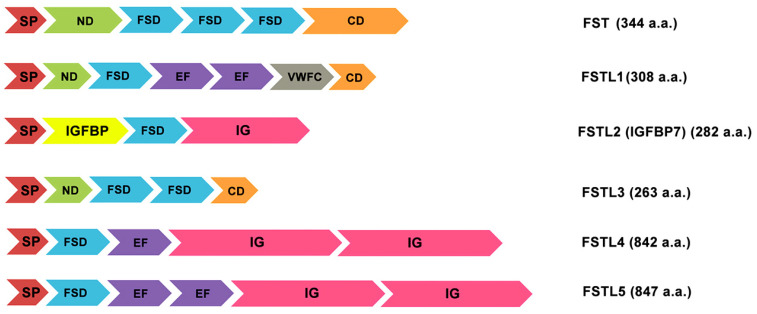
Variety of follistatin-like proteins and their domain organization. SP: signal peptide; ND: N-terminal domain; FSD: Follistatin/Kazal-like domain; EF: a helix-loop-helix structural domain; VWFC: von Willebrand factor type C domain; IGFBP: IGF-binding domain; Ig: immunoglobulin-like domain; CD: C-terminal domain with acidic L-amino acids. The text on the right shows the accepted name of the protein and the number of its constituent amino acids (a.a.) [1,2,30,44,45,46,47].

Considering the features of transcription of follistatin-like proteins, it should be noted that follistatin itself is a glycoprotein of autocrine origin that is expressed in nearly all tissues of mammals (Figure 1). The alternative splicing of the FST gene leads to the formation of two precursors, either 317 or 344 residues in length, which are post-translationally modified to produce mainlyisoforms [35] (Figure 2).

The first and most studied gene *FSTL1* is located on chromosome 3q13.33 in humans and consists of 11 exons. Exons 2 through 11 encode a 308 amino acid protein. The last exon also contains the coding sequence for microRNA-198 (*MIR-198*). Consequently, the FSTL1 primary transcript serves both as mRNA and as pre-miRNA. Besides, the 3 ‘untranslated region of this gene contains several miRNA-binding sites, three of which (miR-206, miR-32-5p, miR-27a) were involved in the regulation of FSTL1 expression [36].

The gene *IGFBP7(FSTL2)*, which contains five exons, is located on chromosome 4q12 in humans. IGFBP7 mRNA is expressed in a wide range of tissues, including parenchymal organs, gastrointestinal tract, brain, heart, placenta, lungs, skeletal muscles, thymus, prostate, testes, ovaries, etc. (Figure 1). Expression of IGFBP7 mRNA and its subsequent translation are regulated by IGF-I, TGF-β and retinoic acid [37]. 

The human follistatin-like protein type 3 (FSTL3) gene also contains five exons, spanning 7004 base pairs (bp) on chromosome 19p13 and giving rise to a main transcript of 2525 bp. The first exon encodes a signal peptide, the second exon corresponds to the N-terminal domain, the third and fourth exons determine the follistatin module and the fifth exon forms the C-terminal region rich in acidic L-amino acids [38,39]. It was found that the process of FSTL3 mRNA transcription is stimulated by transforming growth factor (TGF-beta) according to the principle of negative feedback and by activin-A indirectly through proteins of the SMAD family [39]. In a similar study, it was shown that the cytokine GDF9 could suppress the basal and activin-induced expression of FSTL3 in the culture of granulosa cells [40]. Moreover, the FSTL3 promoter identifies sensitive elements to the transcription factor NF-κB, which also stimulates the expression of *FSTL3* [41]. Noteworthy is the fact that overproduction of FSTL3 mRNA occurs in some cells in response to induced hypoxia. In this case, the hypoxia-inducible factor 1-alpha (HIF1A) seems to play an important role. In other studies, it was shown that phorbol-12-myristate-13-acetate (PMA) and the combination of 17-beta-estradiol with progesterone increased the transcription from the *FSTL3* gene in the culture of human endometrial stromal cells [42].

Recently, new variants of follistatin-like proteins have been discovered: FSTL4 and FSTL5. Now, both these genes and their protein products are characterized much worse than the first three representatives of this large family, which is a certain challenge for the scientific community. It is now obvious that the human gene *FSTL4* is located on chromosome 5q31.1 and consists of 16 exons. Moreover, the last exon of this variant also contains the coding sequence for microRNA (*MIR-1289-2*), as in the case of FSTL1 [39].

FSTL5 was first identified in human brain tissue in the mid-90s. Only 10 years after this event the topology of the gene was studied, and its nucleotide sequence was sequenced. FSTL5 is located in humans on chromosome 4 at locus 4q32.2. The *FSTL5* gene sequence includes 4867 bp, which encodes a large protein with a molecular weight of 93 kDa and is composed of 847 amino acid residues. Five splice variants are described for FSTL4 and FSTL5 [39].

## 3. General Principles of the Structural Organization of Follistatin-Like Proteins

Follistatin and related proteins belong to a wider SPARC family of proteins that share structural and functional similarities [30,31,43,44].

Follistatin-like proteins differ in a similar molecular architecture and domain arrangement as follistatin which itself contains an N-terminal domain and three follistatin domains (FSD1, FSD2, and FSD3) (Figure 2), with a heparin-binding site (HBS) located in FSD1 that promotes binding to extracellular proteoglycans [35]. In addition to signal peptides, domain organization of follistatin-like proteins includes an FSD domain consisting of a cysteine-rich follistatin module (FM) and an evolutionarily conserved Kazal domain (KD), which is characteristic mainly of serine protease inhibitors (Figure 2). The topology of the activin-binding and heparin-binding sites can vary among different representatives of follistatin-like proteins. They can follow either before the FSD, or in the N-terminal region (ND), or after it. Along with the amino-acid-enriched region, the C-terminal part usually contains an immunoglobulin-like domain (Ig) or an area that is homologous to C-like domain of von Willebrand factor (VWFC) (Figure 2) [1,2]. It should be mentioned that these domains of follistatin-like proteins are a characteristic structural element of all proteins of the osteonectin family (SPARC) [30,44,45,46]. 

Despite a specific homology, follistatin-like proteins also have certain differences in the structure of the primary, secondary, or quaternary protein structures. Thus, the structure of FSTL-1 contains a common secretory signal, a follistatin-like domain, a duplicated EF-hand domain, and a VWFC region (Figure 2) [1,2,44]. This protein is found in two differentially glycosylated isoforms with similar functional activity in humans and animals [30,45]. Figure comparison of human and mouse protein sequences shows a high degree of similarity (94.4%) [4,34].

The IGFBP7 (FSTL2) should be considering separately. It is worth noting that this protein is not taken into account as a full-fledged follistatin-like protein. Although IGFBP7 has a sequence similar to the follistatin module and an activin-binding function, the dominant functional domain here is a region capable of binding with a high affinity to insulin-like growth factor (IGF). This protein–protein complex modulates binding of IGF to corresponding receptors playing roles in the promotion of cell proliferation and the inhibition of cell death (apoptosis). Therefore, this protein is better known as IGFBP7 (IGF binding protein 7) or IGFBP-rP1 (IGF binding protein-related protein 1) [47].

Follistatin-like protein 3 (FSTL3) is one of the most controversial members of this family, which is present in cells in a monomeric glycosylated form. It contains two common modules with follistatin and two Kazal-like domains in its structure, besides the required signal sequence (Figure 2). 

In FSTL-4 and -5, structures are found such as Kazal-like domain, only one follistatin-like module, one or two EF-domain and immunoglobulin-like domain, involved in intercellular interactions.

## 4. Follistatin-Like Proteins and Cellular Signaling

**Figure 3:**
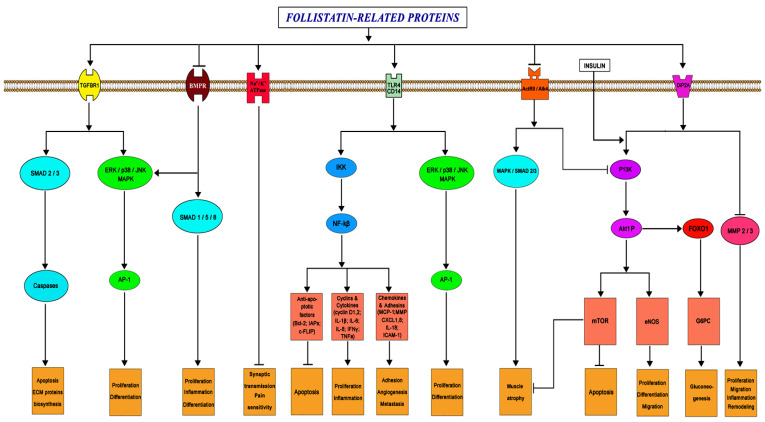
Simplified diagram of the main signaling pathways characteristic of follistatin-like proteins (arrows imply activation of the pathway) [7,10,12,21,22,23,24,25,26,27,28,29,30,31,32,49].

Follistatin is known potently to neutralize activins, members of TGF-β superfamily ligands. In this process, an important role is played by hydrophobic residues within the N-terminal domain that constitute essential activin-binding determinants in the follistatin molecule [38]. On the other hand, this protein does not neutralize BMP receptor interaction, suggesting instead a tripartite complex between ligand, its receptor, and follistatin that might indirectly modulate or even enhance growth factor activity [38]. Follistatin-like proteins, which have a high mutual homology to follistatin, are aimed at interacting with the same pool of receptors (Figure 3). At the same time, the existing features of the primary and spatial structure of these proteins apparently determine the differences in the degree of affinity of the ligand and the receptor, what will be discussed next.

Transgenic models have demonstrated that FSTL1 is involved in a variety of physiological signaling pathways and in a number of pathological conditions [32]. As already mentioned, FSTL1 interacts with numerous receptors of the TGF-β superfamily, among which it is especially necessary to distinguish the type I transforming growth factor beta receptor (TGFBR1) and bone morphogenetic protein receptors (BMP-receptors) [48]. By the way, FSTL1 was initially isolated as TSC-36 (TGF beta stimulated clone 36) [32]. Experiments on coimmunoprecipitation showed that FSTL1 is able to interact with both the BMPRII receptor and the BMP4 factor itself, being its antagonist (Figure 3). It is suggested that FSTL1 interferes with receptor ligands and thereby inhibits BMP signaling.

FSTL1 has also been shown to interact with disco-interacting protein 2 (DIP2A), toll-like receptor 4 (TLR4), and glycosylphosphatidylinositol (GPI)-anchored protein (CD14) (Figure 3) [32,45,49]. FSTL1 activity can be expressed via the NF-kB signaling pathway: FSTL1 induced gene expression of various cytokines and chemokines associated with NF-kB, including IL-1β, TNFa, IL-6, CXCL8/IL-8, and CCL-2/MCP-1 in monocytes and macrophages. FSTL1 is able to induce a pro-inflammatory response by transmitting IKKb–NF-kB signals (Figure 3) [28]. In addition, several supplementary signaling molecules are an essential part of a number of other important pathophysiological processes, including the anti-inflammatory mechanism. Designated molecules may include AKT1, eNOS, some mitogen-activated protein kinases (MAPK), SMAD, and AMPK-activated protein kinase (Figure 3) [45]. Phosphorylation of SMAD transcription modulators is considered one of the ways in which FSTL1 and its homologues regulate the proliferation of certain cell types.

FSTL3 also interacts indirectly through a myostatin and activin-A with activin type 2 receptors (ActRII) (Figure 3). However, unlike FSTL1, FSTL3 is able directly to block myostatin and activin-A and thereby influence the development of myocytes via the SMAD-dependent mechanism [50,51]. 

To date, follistatin-like proteins 4 and 5 are the least studied. Meanwhile, overexpression of FSTL5 significantly increases the level of cleaved activated caspases-3, -8, and -9. As FSTL5 is effectively able to activate important proteins of the caspase pathway, it is believed that apoptosis mediated by FSTL5 may be pronounced caspase-dependent [24]. 

Thus, the degree of involvement of follistatin-like proteins in main steps of cellular signaling indicates an important role performed by these proteins, the full depth of which remains to be discovered by the scientific community. A detailed study of the structure and mechanism of action of follistatin-like proteins will contribute to a better understanding of the features of the development of certain pathological conditions at the molecular level and to the progress in pharmacology and clinical medicine in the future.

## 5. Biomedical Importance of Follistatin-Like Proteins

### 5.1. General Biological Significance

The entire range of functions of proteins, which are studied in this article, is not fully understood. However, it can be argued that they are involved in not only the regulation of physiological processes, such as cell repair, proliferation and differentiation, but in the pathological processes of carcinogenesis and metastasis [4,33,52,53,54,55]. Therefore, it can be argued that follistatin-like proteins are promising targets and biomarkers in practical medicine.

The highest concentration of FSTL-1 is achieved in the heart, lungs, and subcutaneous white adipose tissue under physiological conditions [32,33,34,56]. FSTL1 is normally produced in the epicardium accelerating the growth of cardiac myofibrils and stimulating vascular endothelial growth factor. To the best of our knowledge, FSTL1 is able to participate in the regeneration of the heart muscle in mammals, primarily due to the activation of angiogenic factors. Moreover, a contravention of its expression in mice leads to respiratory failure and death within a few hours after birth [4,10,11,32].

FSTL3 expression has also been found in the heart, placenta, gonads, and pancreas. The most studied functions of this polypeptide are the neutralization of the TGF-β family ligands, in most cases with impact on activin-A and myostatin, as well as the suppression of their activity [57]. This activity has a lot in common with that of follistatin. In addition to this, FSTL3 plays an important role in ovary like the follistatin. FSTL3 and the BMPs are known to be involved in the stimulation of cellular differentiation of the estrogen producing granulosa cells during folliculogenesis within the ovary [40].

Currently, FSTL4 and FSTL5 are ‘in the shadows’, since their biological functions and clinical significance are still poorly understood. Meanwhile, the results of few studies indicate the role of this protein in the process of neoplastic transformation and metastasis [58,59]. It should be emphasized that the highest expression values of these proteins occur in some areas of the central nervous system (Figure 1B).

It is worth mentioning that various pathological conditions, including CVD, cancer progression, and systemic autoimmune diseases, are sometimes directly dependent on the expression of some follistatin-like proteins [9,32,34], which will be discussed below.

### 5.2. Clinical and Diagnostic Importance

#### 5.2.1. Pathologies of the Cardiovascular System

As noted above, FSTL1 is involved in cardiac muscle regeneration in mammals. By acute coronary syndrome and myocardial infarction, the concentration of epicardial FSTL1 transiently increases and returns to normal levels after healing [4]. This information has proved extremely useful in developing innovative approaches to the treatment of cardiovascular complications. Thus, during the application of the collagen “patch”, which was impregnated with recombinant FSTL1, an increase in the concentration of this protein was observed in the affected area of the heart of the model animals. Such manipulations triggered the necessary regeneration processes. This method increased the survival rate in all the studied animals, regardless of the time of applying the “patch” [4,60].

Additionally, recently the effect of FSTL1 injections in cardiac ischemia has been actively studied on the ability to regenerate myocardial cells. Yasuhiro Ogura et al. performed consecutive intravenous injections of FSTL1 in laboratory animals with induced myocardial ischemia in their study. At the same time, there was a rapid growth of muscle fibers and a gradual decrease in the formation of scar tissue. The study found that FSTL1 activated AMP-activated protein kinase (AMPK) and inhibited BMP4. Thus, FSTL1 injections eliminated myocardial damage by inhibiting apoptosis and inflammatory responses [25,61], but patients with chronic heart failure had a high expression of FSTL1, which returned to normal after therapy [28]. This condition may have been associated with vascularization in the affected area, since the expression level of FSTL1 correlates with that of the endothelial cell marker CD31 [5]. 

Thus, it is obvious that FSTL1 protein with autocrine antihypertrophic signaling mediated by AMP-activated protein kinase has a whole range of physiological properties, the most important of which are protective and regenerative abilities [32,62,63,64,65]. The injection of FSTL1 into the body prevents extensive damage to the heart and malignant vascular remodeling. The deficiency of FSTL1 aggravates cardiovascular pathologies. FSTL1 affects multiple cell-type-specific signaling pathways. Simultaneously, there are data indicating that the severity of various effects may be in a certain dependence on the degree of glycosylation of this protein [32,62,63,64,65,66,67,68]. 

An interesting fact is that FSTL1 has been shown to affect the function of the Na^+^/K^+^ ATPase channel (Atp1a1) (Figure 3). Atp1a1 maintains the cell membrane potential that is critical for nerve impulse conduction in the synaptic system and for the normal functioning of the cardiac conduction system [63].

FSTL3 also plays an important and sometimes controversial role in the cardiovascular system. This protein is activated in heart failure and returns to normal after myocardial recovery [5,7]. The level of FSTL3 increases in heart failure of various etiologies and correlates with some markers of the severe stage of the disease (skeletal α-actin and natriuretic protein (BNP)) [5]. The level of FSTL3 was usually determined in relative units as the change in expression levels of mRNA of target gene in relation to internal reference genes using quantitative real-time RT-PCR. An increased concentration of FSTL3 or their mRNA are often observed in myocardial hypertrophy, preventing the antihypertrophic effects of activin-A, as FSTL3 is its inhibitor [5,8,69,70].

#### 5.2.2. Pathologies of the Respiratory System

FSTL1 influences lung tissue as it is expressed in most types of mesenchymal cells [12]. A decrease in FSTL1 in mice has been shown to result in respiratory failure leading to postnatal mortality. Moreover, inactivation of the *FSTL1* gene leads to a contravention of the formation of the cartilaginous base of the respiratory tract. This is manifested by a stop in development and a decrease in the total number of formed semicircles of the tracheal trunk [71]. According to some data, FSTL1 is necessary not only for the formation of tracheal cartilage, but also for the processes of alveolar maturation [11,12]. 

Proteomic analysis of sputum from patients with asthma showed that FSTL1 is one of the most expressed proteins [32,72,73]. In addition, the level of FSTL1 production correlates negatively with lung function parameters and positively with markers of airway remodeling [32].

It has also been demonstrated in mouse models that FSTL1 acts as a reliable BMP-4 antagonist in the processes that control the development of lung tissue [10,11,12,71]. FSTL1 regulates some important signaling pathways, such as BMP, SHH, WNT, and FGF. This regulation is observed in the development of lung smooth muscle and related diseases, including pulmonary artery hypertension [11,74,75].

It was also found that laboratory mice showing low FSTL1 expression parameters were prone to spontaneous development of emphysema. Microcomputer tomography scans revealed that these mice had increased lung volume and decreased lung density. Collectively, these data indicate that a decrease in the FSTL1 expression was a sufficient condition for the development of a histological, functional, and radiological picture of emphysema [76].

FSTL3 is extensively expressed by human respiratory epithelial cells at both the RNA and protein levels. FSTL3 is capable of binding the activin-A protein and is involved in many inflammatory processes in the body. Normally, FSTL3 performs a protective role, preventing airway remodeling. In some patients with asthma, a deficiency in the epithelial expression of FSTL3 leads to an increase in the proportion of activin-A and, consequently, to the activation of airway fibroblasts and further progression of fibrosis [13,14,77].

#### 5.2.3. Carcinogenesis

The participation of follistatin-like proteins has been noted in the processes of tumor development and metastasis. The expression of FSTL1 is reduced in various types of cancer, such as cancer of the prostate, ovary, endometrium, pancreas, kidney, nasopharyngeal carcinoma and lung adenocarcinoma in comparison with healthy tissue [17,18,19,23,32,44,78,79,80,81,82,83,84,85,86]. It should be underlined that FSTL1 plays a key role in lung organogenesis; however, its activity decreases during the development and progression of lung cancer. Decreased levels of FSTL1 have been found in various tumors compared to normal tissues and have been associated with poor clinical prognosis in patients with ovarian cancer, non-small cell lung cancer, especially with lung adenocarcinoma [20,32,78]. 

Contrarily, increased expression of FSTL1 as opposed to healthy tissue was observed in brain cancer, castration-resistant prostate cancer, in most cases of hepatocellular carcinoma (HCC), squamous cell carcinoma of the head, neck, and esophagus [32,79,83].

To date, the complexity and variety of mechanisms underlying tumor development do not allow us to form a complete picture of the role of FSTL1 in the development and progression of cancer. Apparently, the tissue tropism of the tumor cell line is the determining factor in the different, sometimes opposite effect of FSTL1.

Numerous studies have shown that changes in the expression of IGFBP7 (FSTL2) are also observed in various types of cancer, including HCC, breast cancer, gastrointestinal cancer, prostate cancer, and many others. It has been suggested that IGFBP7 acts primarily as a tumor suppressor, since its expression is reduced in neoplastic tissues of various types of cancer, including liver tumors [87]. However, subsequent studies did not support this hypothesis. For instance, in a recently published article Huang et al. showed that the level of serum IGFBP7 in esophageal squamous cell cancer cells is consistently higher than in healthy cells. This can serve as a potential biomarker in diagnosis [88].

FSTL3 is also involved in the regulation of tumor development [21,22,89,90]. For example, upregulation of FSTL3 has been observed in invasive breast cancer. Overexpression of this protein may promote the proliferation of tumor cells due to the antagonism of endogenous activators [21]. FSTL3 expression is increased in non-small cell lung cancer. Such overexpression of FSTL3 may promote proliferation and metastasis in this type of cancer, while knockdown of FSTL3 plays the opposite role [22].

Previous studies have clearly shown that the FSTL5 protein is suppressed in HCC cells, while its expression correlates with a good prognosis in patients. In vitro and in vivo results demonstrate inhibition of HCC growth by FSTL5. It is pointed out that a similar effect is possible by stimulating caspase-dependent apoptosis of HCC cells and regulation of proteins of the Bcl-2 family, bypassing the direct effect on the cell cycle. By contrast, FSTL5 correlates with a poor prognosis in medulloblastoma and colorectal cancer [58,59]. Therefore, FSTL5 could be a potential target for the treatment and prognosis of several types of cancer. However, a more detailed interpretation of the molecular mechanism requires further research and data systematization [24].

#### 5.2.4. Inflammatory Process

The role of FSTL1 in inflammatory processes has been studied in several laboratory models and can be as pro-inflammatory as anti-inflammatory [32,91]. 

High FSTL1 levels are found in many systemic autoimmune diseases, such as rheumatoid arthritis, osteoarthritis, and Sjogren’s syndrome [26,28,29,32,92].

The functions of FSTL1 during the inflammatory process are dual. During the acute period, these proteins act as an anti-inflammatory factor, but they have a pro-inflammatory effect in chronic diseases. This can be explained by the activation of various signaling pathways. Initially, FSTL1 binds the DIP2A receptor and prevents the degradation of tissue proteins by matrix metalloproteinases (MMP) (Figure 3). Subsequently, FSTL1 activates the inflammatory response via the TLR4/CD14 pathway, activates the AMPA pathways, and inhibits the BMP signaling pathways [4,23,32,93,94]. However, additional endogenous or exogenous factors, which may be involved in the regulation, should not be excluded.

For instance, association of the severity of rheumatoid arthritis with the ability of FSTL1 to activate the signaling pathways MAPK, JAK/STAT3, and NF-kB and increase the secretion of metalloproteinases 1, 3, and 13 was shown in the article of Ni et al. [95]. At the same time, the concentration of FSTL1 in the biological fluids of patients with rheumatoid arthritis is positively correlated with the severity of the disease. Therefore, this protein can be considered simultaneously as a diagnostic marker and a promising pharmacological target for the prevention and treatment of complications of rheumatoid arthritis.

The FSTL3 level in patients with autoimmune diseases correlated with the scale of the severity of radiological changes in rheumatic diseases (Kellgren–Lawrence scale) [96,97]. 

Circulating FSTL3 is associated with renal function in both patients with chronic nephritis and patients with acute renal dysfunction. Recently, FSTL3 was hypothesized to be eliminated by the kidneys and might counteract adverse activin/myostatin signaling observed in renal dysfunction [98].

It is worth noting that an increase in the concentration of FSTL3 is observed in the blood serum and placenta in a serious multisystem pathological condition such as preeclampsia, which occurs in the second half of pregnancy. Meanwhile, the degree of FSTL3 involvement in the pathogenesis of this disease is still dubious [27].

#### 5.2.5. Pathologies of Osteo- and Myohistogenesis

Since FSTL1 is a BMP inhibitor, changes in its concentration affect the development of bone tissue pathologies. Complete inactivation of FSTL1 leads to skeletal defects in the embryonic development of mice. Deletion of the *FSTL1* gene resulted in both decrease in the size of the bones and their incorrect location [10].

Considering the above mentioned, an interesting fact is the ability of FSTL3 to suppress osteoclast differentiation by binding to disintegrin and metalloproteinase-12 (ADAM12), modulate insulin sensitivity, influence adipose tissue homeostasis, and bind BMP proteins. Apparently, FSTL3 is directly involved in the formation and strengthening of bone tissue during physical activity. Exercise increases the expression of FSTL3 mRNA and protein in osteoblasts, and deletions of the *FSTL3* gene lead to a loss of mechanosensitivity, mechanical strength, and exercise-induced bone formation. It should be mentioned that FSTL3 regulates muscle mass, since it is an antagonist of myostatin which is a muscle growth inhibitor [50].

Results from a recent study associate variation of FSTL5 expression in prehypertrophic chondrocytes with lower limb malformations and congenital rotational foot deformity [99].

#### 5.2.6. Pathologies of Lipid and Carbohydrate Metabolism

FSTL1 is actively expressed in adipose tissue by preadipocytes and adipocytes. This protein induces inflammatory reactions in adipocytes and macrophages and suppresses insulin signaling in adipocytes [100,101]. Martin Horak et al. showed in their study that overweight and moderate obesity are associated with increased levels of FSTL1 primarily due to its pro-inflammatory action during chronic mildly inflammatory condition [102]. On the other hand, pathological and severe obesity decreased FSTL1 levels due to a continuous increase in the number of matured adipocytes and other associated factors [102,103,104]. In addition, the secretion of FSTL1 may be regulated by hyperinsulinemia and free fatty acids [105,106,107].

FSTL3 levels are also increased in obese patients and are positively correlated with total fat mass and the amount of the hormone leptin secreted by adipocytes from adipose tissue. FSTL3 levels increase in response to TNF-a, but not IL-6. This suggests that TNF-a is an inflammatory factor that determines the increase in systemic levels of FSTL3 [57,108,109].

Experimental knockout of the *FSTL3* gene did not lead to a noticeable decrease in muscle mass, or the cross-sectional area of muscle fibers, but significantly increased glucose tolerance and enhanced insulin sensitivity. This is probably due to in the increased pancreatic island number and size, suggesting a role of FSTL3 in glucose homeostasis [108]. In addition, it is likely that the effect of some follistatin-like proteins on glucose levels may be FoxO1-dependent (Figure 3).

Thus, adipose tissue and glucose level can make a significant contribution to the regulation of FSTL3. This suggestion is confirmed by the fact that women with a higher percentage of fat and an increased level of leptin have high expression of this protein [110,111,112,113,114].

#### 5.2.7. Pathologies of Central Nervous System

FSTL1 plays a role in the regulation of synaptic transmission and pain threshold. It is secreted by sensory afferent axons. FSTL1 activates the presynaptic α1 subunit of Na^+^/K^+^-ATPase, which leads to membrane hyperpolarization and inhibition of neurotransmitter release. Loss of FSTL1-dependent activation of Na^+^/K^+^-ATPase leads to increased synaptic transmission and sensory hypersensitivity (Figure 3) [31,45,115]. FSTL1 is activated during neuroinflammation, while an increased FSTL1 level correlates with a decrease in the concentrations of proinflammatory cytokines (TNF-α, IL-1b, and IL-6) [26,116]. However, FSTL1 can also play a dual role in the inflammatory process in the central nervous system: pro- and anti-inflammatory [26,31,45,115,117].

The pro-inflammatory effect of FSTL1 is only a small step in the overall mechanism in which this protein also exerts a powerful anti-inflammatory effect through the activation of the MAPK/pERK1/2 signaling pathway at an early stage of inflammation [26]. Further study of the signaling pathways involved in the expression of FSTL1, as well as their cross-interactions, may provide a potentially new therapeutic approach to the treatment of certain neuropathic diseases.

Interestingly, the level of expression of the *FSTL5* gene is higher in the brain and spinal cord compared to other organs. A more detailed study shows limited expression of the *FSTL5* gene in the olfactory system. Based on this fact, FSTL5 may be considered to be involved in the perception and processing of olfactory information [118]. Therefore, a detailed study of FSTL5 expression in pathological processes of the central nervous system will provide a better understanding of the mechanism of their occurrence and development.

## 6. Conclusions

The expression of follistatin-like proteins is observed in almost all organs and systems of the human and animal body. Widespread distribution of these proteins may have both positive and negative effects.

First, it is necessary to highlight the positive effect of FSTL1 on the processes of regeneration of the heart muscle, which may have prospects not only in the diagnostics, but also in the creating effective drugs based on this protein in the future. In addition, further study of the role of FSTL1 and IGFBP7 in the development of inflammatory processes and cell malignancy opens up prospects for their practical utilization. For example, they can be used as pharmacological agents in various pathological conditions, such as obesity, neuroinflammation and some types of cancer.

FSTL3 can find application in pathologies of the musculoskeletal and respiratory systems due to its ability to bind to representatives of the TGF-β superfamily such as myostatin and activin-A. Besides, the level of FSTL3 expression itself can act as a diagnostic biomarker reflecting the dynamics of bone tissue formation in healthy people and patients with metabolic disorders. A better understanding of the role of activin-A/FSTL3 signaling in lung fibrosis may lay the foundations for new therapeutic approaches to the treatment and prevention of airway remodeling in asthma and other bronchopulmonary diseases. 

FSTL5 is also capable of firmly entering medical practice as a potential target and predictive molecular marker for the diagnosis, treatment and prognosis of HCC and colorectal cancer. Overexpression of FSTL5 positively correlated with a good prognosis in HCC patients, since it reliably inhibited the growth of HCC by stimulating caspase-dependent cell apoptosis and regulating proteins of the Bcl-2 family.

To sum up, follistatin-related proteins and their encoding gene sequences can be designated not only as informative diagnostic biomarkers but also as promising candidates for the development of new methods of treatment for a number of socially significant diseases.

## Data Availability

Data sharing not applicable.
